# iTRAQ Quantitative Proteomic Analysis of Vitreous from Patients with Retinal Detachment

**DOI:** 10.3390/ijms19041157

**Published:** 2018-04-11

**Authors:** Fátima Milhano Santos, Leonor Mesquita Gaspar, Sergio Ciordia, Ana Sílvia Rocha, João Paulo Castro e Sousa, Alberto Paradela, Luís António Passarinha, Cândida Teixeira Tomaz

**Affiliations:** 1 CICS-UBI—Health Sciences Research Centre, University of Beira Interior, 6201-506 Covilhã, Portugal; ftxsantos@gmail.com (F.M.S.); leonormgaspar@gmail.com (L.M.G.); asilviarocha@gmail.com (A.S.R.); jpcastrosousa@netcabo.pt (J.P.C.e.S.); lpassarinha@fcsaude.ubi.pt (L.A.P.); 2 Chemistry Department, Faculty of Sciences, University of Beira Interior, 6201-001 Covilhã, Portugal; 3 Laboratory of Pharmacology and Toxicology—UBIMedical, University of Beira Interior, 6200-284 Covilhã, Portugal; 4 Unidad de Proteomica, Centro Nacional de Biotecnología, CSIC, Calle Darwin 3, Campus de Cantoblanco, 28049 Madrid, Spain; sciordia@cnb.csic.es (S.C.); Alberto.Paradela@cnb.csic.es (A.P.); 5 Hospital Center Leiria-Pombal, 3100-462 Pombal, Portugal

**Keywords:** iTRAQ, quantitative proteomics, retinal detachment, vitreous proteome

## Abstract

Rhegmatogenous retinal detachment (RRD) is a potentially blinding condition characterized by a physical separation between neurosensory retina and retinal pigment epithelium. Quantitative proteomics can help to understand the changes that occur at the cellular level during RRD, providing additional information about the molecular mechanisms underlying its pathogenesis. In the present study, iTRAQ labeling was combined with two-dimensional LC-ESI-MS/MS to find expression changes in the proteome of vitreous from patients with RRD when compared to control samples. A total of 150 proteins were found differentially expressed in the vitreous of patients with RRD, including 96 overexpressed and 54 underexpressed. Several overexpressed proteins, several such as glycolytic enzymes (fructose-bisphosphate aldolase A, gamma-enolase, and phosphoglycerate kinase 1), glucose transporters (GLUT-1), growth factors (metalloproteinase inhibitor 1), and serine protease inhibitors (plasminogen activator inhibitor 1) are regulated by HIF-1, which suggests that HIF-1 signaling pathway can be triggered in response to RRD. Also, the accumulation of photoreceptor proteins, including phosducin, rhodopsin, and s-arrestin, and vimentin in vitreous may indicate that photoreceptor degeneration occurs in RRD. Also, the accumulation of photoreceptor proteins, including phosducin, rhodopsin, and s-arrestin, and vimentin in vitreous may indicate that photoreceptor degeneration occurs in RRD. Nevertheless, the differentially expressed proteins found in this study suggest that different mechanisms are activated after RRD to promote the survival of retinal cells through complex cellular responses.

## 1. Introduction

Retinal Detachment (RD) is a potentially blinding disease characterized by a physical separation between the neurosensory retina (NSR) and the underlying retinal pigment epithelium (RPE) [1,2]. Modifications in adhesion between the NSR and RPE and the degradation of interphotoreceptor matrix glue can be involved in the onset of RD [3,4]. Risk factors such as age, the level of oxygenation, and other ocular diseases (e.g., myopia, vitreoretinal degeneration) contribute to reducing the retinal adhesion, and therefore to the development of RD [3,4,5]. The most common type of RD is rhegmatogenous, with an incidence of 6.3–17.9 per 100,000 people per year. Its pathogenesis is manifested by the presence of a full-thickness retinal break [1,4,6]. Rhegmatogenous retinal detachment (RRD) may be triggered by vitreous syneresis, a liquefaction of gel caused by age or by trauma, which reduces the vitreoretinal adhesion and results in deflation and relaxation of the collagen network and in the accumulation of vitreous fluid in subretinal space [3,4,7]. Subsequently, vitreous falls upon itself causing the physical separation between the NSR and the RPE of the retina, leading to severe and permanent loss of vision [4,5,7]. 

The treatment of RD has dramatically improved over the past decades. Surgical procedures including scleral buckling, pars plana vitrectomy, and pneumatic retinopexy have been successfully used for the treatment of RRD, with primary success rates of up to 90% [6,8,9]. However, when RRD is associated with macular detachment, choroidal detachment (RRDCD) or PVR, the patients experience poor visual recovery and low reattachment rates [10,11]. Besides the structural changes that occur in the retina, the complex biomolecular mechanisms that are activated following RRD can also play an important role in its pathogenesis. As matter of fact, numerous cytokines, and pro-inflammatory and growth factors are released in vitreous after RD. It was proposed that these molecules have a relevant role in the reparative wound-healing process and retinal photoreceptor apoptosis in RRD, and consequently, may improve the post-surgical visual outcomes [12]. Also, the proteome and biochemical properties of vitreous are directly affected by physiological and pathological conditions of the retina [13,14,15]. So, vitreous is a suitable matrix for studying the pathophysiological mechanisms in the RRD. 

Quantitative proteomics provides an additional approach to understand the global proteomic dynamics by identifying and comparing quantitatively several proteins in ocular fluids [16,17]. Although the application of proteomics technology in ophthalmic research is becoming increasingly common [15,18,19], the published information about vitreous proteome in RD is scarce. Indeed, the majority of these studies are focused on PVR [14,20,21,22] with the application of different proteomic techniques. In the current study, isobaric tags for relative and absolute quantitation (iTRAQ) labeling was combined with two-dimensional LC-ESI-MS/MS (2DE-LC-MS/MS) to find expression changes in the proteome of vitreous from patients with RRD when compared to macular epiretinal membranes (MEM). This work was focused in RRD, the most common but less severe type of RD, in order to understand the complex biological processes that are activated after RD.

## 2. Results

### 2.1. Characterization of Patients and Vitreous Samples

Demographic and clinical characteristics of patients enrolled in the study and the description of the corresponding vitreous samples are summarized in Table 1. The study groups consisted of 15 patients, 9 women, and 6 men, with ages comprised between 52 and 84 years. The RRD group included 8 patients and the control group was composed of 7 patients with MEM. From these, 8 patients were selected for the analysis of the differentially expressed proteins using iTRAQ-based analysis. Specifically, vitreous collected from 4 patients (1 male, 3 females) with RRD were analyzed and compared to the vitreous collected from 4 patients (2 males, 2 females) with MEM. In the RRD group, the 4 patients had macula-off RRD, with an extension of detachment of 2 (n = 2), 3 (n = 1) and 4 (n = 1) quadrants. Seven patients were selected for the validation of protein biomarkers by Western blotting (WB), 4 with RRD (2 males, 2 females) and 3 with MEM (1 male, 2 females). From these patients, 3 had macula-in RRD and 1 macula-off RRD, with an extension of detachment of 1 (n = 3) and 3 (n = 1) quadrants. Both groups were similar in age and gender, but the patients with RRD had a lower median age, 64 ± 7 years, compared to a median of 76 ± 5 from the patients of the control group. The protein concentration was slightly higher in vitreous from patients with RRD, averaging 3.12 ± 2.96 µg/µL, than in MEM group, with average concentrations of 2.66 ± 1.63 µg/µL. In patients with RRD, the total protein concentration in vitreous increases with the extension of RD (number of quadrants) and in the macula-off RRD.

### 2.2. Vitreous Proteome in Rhegmatogenous Retinal Detachment (RRD)

By combining iTRAQ labeling with 2D-nano-LC-MS/MS, 1030 proteins were identified with 6078 peptides, of which 2613 correspond to unique peptides (Supplementary Table S1). To recognize which proteins were newly found in the present study, the identified proteins were compared to previous vitreous proteomics reports [19,23,24,25,26,27,28,29,30,31,32,33,34,35], as seen in Supplementary Table S2. 

From the identified proteins, 150 were found differentially expressed in RRD versus MEM, including 96 overexpressed and 54 underexpressed (Supplementary Table S3). In literature, iTRAQ ratios >1.2 (overexpressed) or <0.82 (underexpressed), with a *p*-value < 0.01, were considered significant fold changes in terms of protein expression [20]. In this study, iTRAQ ratios between 221.22 and 2.06 were reported for the overexpressed proteins, and iTRAQ ratios between 0.00 and 0.52 were reported for the underexpressed, with *p*-values below 0.01 (Supplementary Table S3). As shown in Table 2, some plasma proteins were significantly decreased in RRD, including retinol-binding protein 4 (RBP4) and apolipoprotein A-IV (APOA4). Proteins displaying highest overexpression include photoreceptor proteins, such as phosducin (PDC), rhodopsin (RHO), and s-arrestin (SAG).

**Figure 1:**
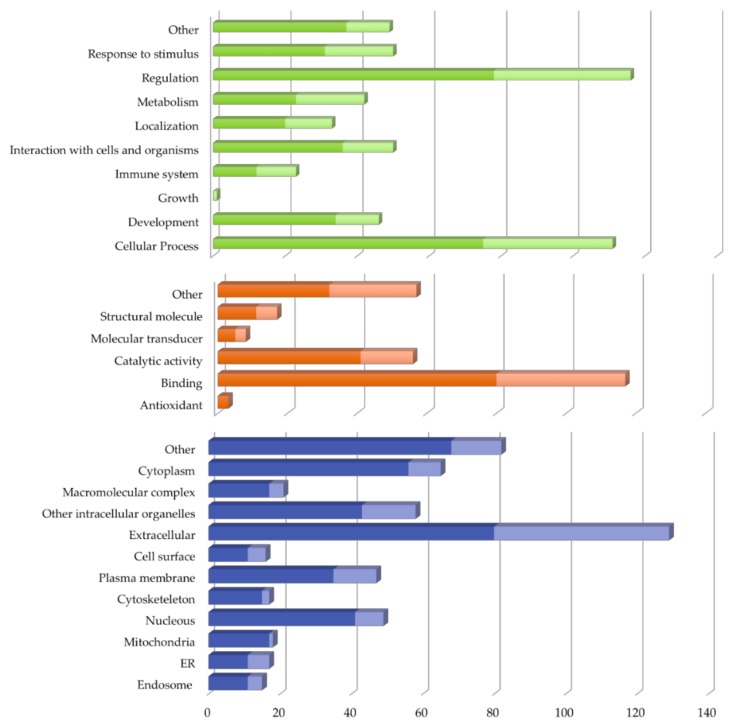
Classification of the 150 proteins found differentially expressed in vitreous of patients with rhegmatogenous retinal detachment in comparison with macular epiretinal membranes samples according to Gene Ontology (GO) terms using STRAP 1.5. GO annotation for biological process, molecular function, and cellular component are represented by green, orange, and blue bars, respectively.

The 150 proteins found differentially expressed in vitreous of RRD compared to MEM were classified according to the related GO (Gene Ontology) terms for biological process, molecular function and cellular component, using STRAP (Software Tool for Rapid Annotation of Proteins), as seen in Figure 1 and in Supplementary Table S4. According to STRAP classification for biological processes (Figure 1), the differentially expressed proteins in RRD vitreous were related to regulation (n = 116) or cellular processes (n = 109). Biological processes, such as signal transduction, apoptosis, cell proliferation, gene expression, and/or RHO mediated signaling pathway were primarily regulated by the proteins found overexpressed in RRD. The regulation of complement activation was found to be mediated by proteins underexpressed in RRD. Regarding cellular processes, various proteins (19 overexpressed, 2 underexpressed) were involved in neutrophil degranulation. A significant part of differentially expressed proteins also participates in metabolic processes, such as gluconeogenesis or proteolysis. Regarding the analysis of molecular function (Figure 1), both overexpressed and underexpressed proteins were mainly binding proteins (n = 117) or/and with catalytic activity (n = 56). Moreover, many of the differentially expressed proteins in RRD were extracellular matrix structural constituents, and a significant part of overexpressed proteins were identified as structural constituents of the eye lens. The categorization according to cellular component (Figure 1) showed that these proteins were largely found in extracellular space (n = 129) and are classified as extracellular matrix components (n = 21) or as blood particles (n = 25). On the other hand, differentially expressed proteins were also localized intracellularly, namely in the cytoplasm (n = 65), nucleus (n = 49), plasma membrane (n = 47), and in other intracellular organelles (n = 58). 

**Figure 2:**
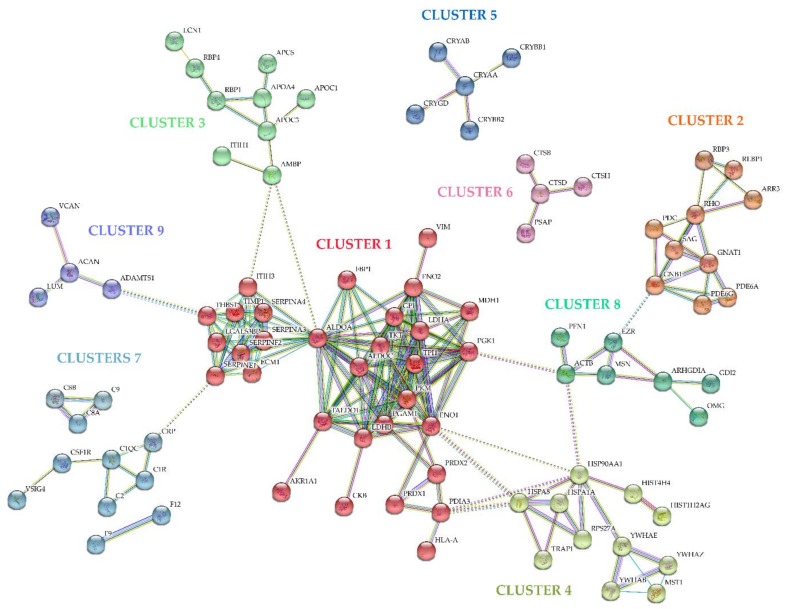
Protein-protein interaction network of the proteins found differentially expressed in RRD, based on interaction evidence, predicted using STRING 10. The protein-protein interaction network (PPI) network was grouped into 11 relevant protein clusters using the ECM clustering option provided by STRING.

Further analyses were made by STRING database (Search Tool for the Retrieval of Interacting Genes/Proteins) to generate an overall protein-protein interaction network (PPI) based on interaction evidence, with high confidence (0.70). The network is enriched in 231 interactions between the 150 proteins found differentially expressed in RRD, with a PPI enrichment *p*-value < 1.0 × 10^−16^. The PPI network was grouped into 11 relevant protein clusters using the Markov Cluster Algorithm (MCL) clustering option provided by STRING, as shown in Figure 2 and Supplementary Table S4. Many of the clusters share interactions among them, indicating that these molecules play key roles in diverse pathways. To infer the functional associations, the clusters were classified according to Reactome and Kyoto encyclopedia of genes and genomes (KEGG) (Supplementary Table S4). 

Cluster 1 (red) is the larger and is associated with carbon metabolism (glycolysis/gluconeogenesis and pentose phosphate pathway), biosynthesis of amino acids, and transcriptional regulator hypoxia-inducible factor-1 (HIF-1) signaling pathway. Carbon metabolism proteins were found overexpressed in this study, including isomerases (TPI1, GPI), aldolases (TALDO1, ALDOA, ALDOC), and other proteins, such as AKR1A1, phosphoglycerate mutase 1 (PGAM1), fructose-1,6-bisphosphatase 1 (FBP1), and phosphoglycerate kinase 1 (PGK1). Glycolytic enzymes (ALDOA, ENO2, and PGK1), metalloproteinase inhibitor 1 (TIMP1), and plasminogen activator inhibitor 1 (SERPINE1) are related to HIF-1 signaling pathway.

Cluster 2 (orange) represents proteins specifically involved in phototransduction, including PDC, RHO, SAG, retinaldehyde-binding protein 1 (RLBP1), retinal phosphodiesterase subunits (PDE6A, PDE6G) and transducin subunits (GNAT1, GNB1). It is important to evidence that high levels of these specific proteins were found in RRD, with iTRAQ ratios between 3.45 and 221.22. Likewise, other proteins essential for eye function can be found in other clusters. In cluster 3 (light green), in which are included plasma apolipoproteins (APOA4, APOC2, APOC3), the only overexpressed proteins are retinol-binding proteins (RBP1, RBP3) and retinaldehyde-binding protein 1 (RLBP1), related to retinoid metabolism and transport, and consequently, to phototransduction. The custer 5 (dark cyan) is composed of the structural components of the lens, alpha-crystallins (CRYAA, CRYAB), and beta-crystallins (CRYBB1, CRYBB2), also overexpressed in RRD.

The Cluster 4 interactions (olive) showed a significant enrichment to PI3K-Akt signaling pathway, Hippo signaling pathway, and HSF1 activation. So, upregulated proteins such as heat shock proteins (HSPA1A, HSP90AA1, and TRAP1), 14-3-3 proteins (YWHAE, YWHAB, and YWHAZ), and Ubiquitin-40S ribosomal protein S27a (RPS27A) are related to cellular responses to heat stress and the regulation of apoptotic signaling. Cathepsins (CTSB, CTSD, and CTSH) and prosaposin (PSAP) are lysosomal enzymes, grouped into cluster 6 (pink), and found overexpressed in RRD vitreous proteome when compared with MEM samples. Proteins involved in complement and coagulation cascades pathways and defense response were grouped into three clusters, denominated clusters 7, colored with cyan. Complement components (C1R, C2, C8A, C8B, C9), coagulation factors (F9, F12), and C-reactive protein (CRP) were found underexpressed in RRD but macrophage colony-stimulating factor 1 receptor (CSF1R), v-set and immunoglobulin domain-containing protein 4 (VSIG4), and complement C1q subcomponent subunit C (C1QC) were found overexpressed. Cluster 8 (see green) is mainly composed of components of myelin sheath, some of them involved in the regulation of actin cytoskeleton. The proteins from cluster 9 (purple) are proteinaceous components of extracellular matrix that participate in glycosaminoglycan biosynthesis and turnover.

### 2.3. Protein Validation by Western Blotting

**Figure 3:**
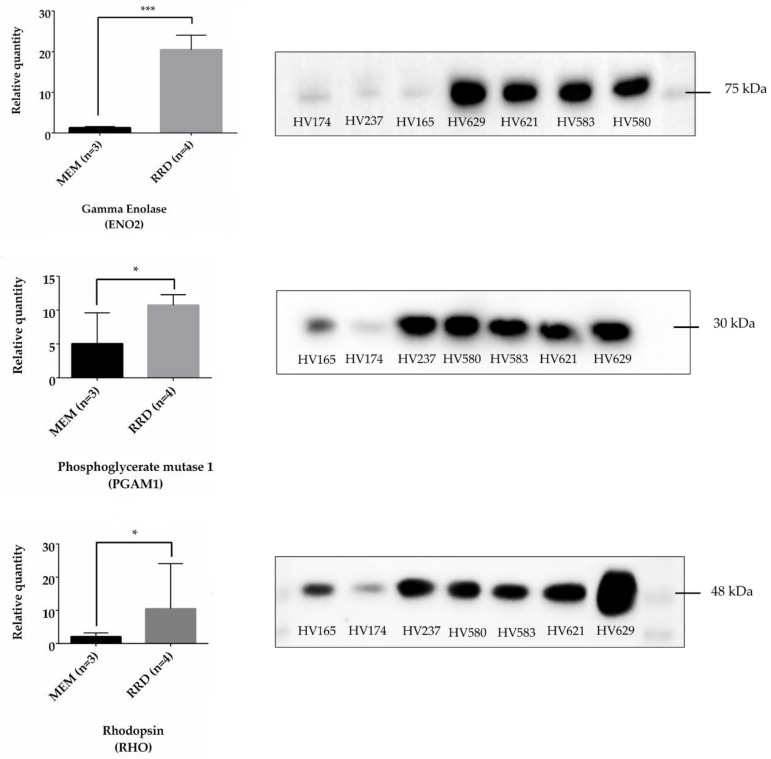
Western blot analyses of ENO2, PGAM1, and RHO in vitreous samples from patients with MEM (HV165, 174 and 237) and RRD (HV580, 583, 621 and 629). Statistics analysis were performed using Mann–Whitney U test, with * and *** representing *p* < 0.05 and *p* = 0.0007, respectively.

For the validation of quantitative results, WB analysis was performed to confirm the overexpression of some proteins in RRD vitreous. Thus, ENO2, PGAM1, and RHO were randomly chosen and detected in RRD (n = 4) and MEM (n = 3) vitreous samples by WB analysis. Changes in protein abundance were highly consistent with the results obtained using iTRAQ (Figure 3). Mann–Whitney *U* test showed a highly significant increase in the levels of a native form of ENO2 (78 kDa) in RRD versus MEM, and this difference is consistent among samples of the same study group. Analysis of PGAM1 and RHO also confirms that these proteins are overexpressed in RRD, but the difference is less significant (*p* < 0.05). The expression of PGAM1 and RHO in the vitreous of patient HV237 (MEM) is similar to the RRD group. RHO is also highly expressed in vitreous of patient HV 629 when compared to the other RRD samples. 

## 3. Discussion

In recent years, effort has been made for the characterization of the complete vitreous proteome, either through analysis of post-mortem samples or samples obtained by vitrectomy. Recently, Loukovaara and co-workers identified the larger set of proteins so far found in human vitreous, using MS-based label-free quantitative proteomics analysis [5]. Indeed, many authors have contributed to the enrichment of our knowledge about human vitreous proteome, [1,2,3,4,5,6,7,8,9,24,25,26,27,28,29] proving that no individual technology can cover completely this proteome. In the present study, 1030 proteins were identified with 6078 peptides, of which 2613 correspond to unique peptides. These proteins were compared to previous vitreous proteomics reports (Supplementary Table S2), including twelve studies in which vitreous were collected by pars plana vitrectomy [19,23,24,25,26,27,28,29,30,31,32,33,35] and one study in which vitreous core was aspirated from post-mortem healthy eyes [34]. Table 3 displays the total number of proteins identified in the vitreous using distinct experimental set-ups, including the number of proteins found exclusively in each study. Most of the identified proteins (808 proteins) have been previously described in vitreous proteome, establishing the validity of the data from the current study. To the best of our knowledge, 222 of the identified proteins were exclusively found in this study, compared to previous reports [19,23,24,25,26,27,28,29,30,31,32,33,34].

Few studies have been published regarding the vitreous proteome in RD and the majority of them were focused in PVR, one of the most common causes of failure to correct RRD [14,20,21,22]. Shitama and colleagues found higher expression levels of pigment-epithelium derived factor and apolipoprotein A1 in RD, compared to other ocular diseases [14]. Yu and colleagues found 516 proteins in vitreous of RRD patients with PVR using SDS-PAGE and reversed-phase liquid chromatography-tandem mass spectrometry [22]. iTRAQ combined with liquid chromatography-electrospray ion trap-mass spectrometry-mass spectrometry (LC-ESI-MS/MS)was used by Wu and co-workers to identify 103 proteins differentially expressed, including 54 up-regulated and 49 down-regulated proteins, in RRDCD when compared with RD [11]. More recently, our research group identified 127 proteins in vitreous of RD patients, of which 68 had not yet been found in previous studies, by combining ion exchange chromatography, SDS-PAGE and MALDI-TOF/TOF analysis [19]. In the current study, iTRAQ labeling was combined with 2DE-LC-MS/MS to find expression changes in the proteome of vitreous from patients with RRD, the most common type of RD. Using this technique, 150 proteins (96 overexpressed and 54 underexpressed) were found differentially expressed in these patients. WB analysis confirmed that the levels of ENO2, PGAM1, and RHO were up-regulated which is consistent with the iTRAQ-based proteomics results. Functional enrichment analyses of the differentially expressed proteins were also applied using STRAP and STRING to better elucidate the molecular mechanism underlying RRD pathogenesis.

Carbon metabolism is the most basic aspect of life since it comprises various pathways essential to obtain energy for cell function and survival. In this study, many of the proteins found differentially expressed in RRD are related to carbon metabolism and to glycolysis. Proteins such as TPI1, GPI, ALDOA, ALDOC, AKR1A1, PGAM1, ENO2, FBP1, PGK1, L-lactate dehydrogenase, pyruvate kinase, as well as the solute carrier family 2, facilitated glucose transporter member 1 (SLC2A1) were found overexpressed. Glycolytic enzymes may be upregulated in RRD in an effort to obtain more energy through glycolysis to compensate the metabolic “stress” state of the retina. Indeed, the function and maintenance of retinal cells require high levels of energy in the form of ATP that are mainly generated from glucose by both anaerobic and aerobic glycolysis [36,37]. Mandal and co-workers already found increased levels of α-enolase in retinal extracts after RD in rabbits, suggesting an upregulation of the glycolytic process, but ALDOA were found underexpressed in that study [38]. Additionally, pentose phosphate pathway, fructose and mannose metabolism, and biosynthesis of amino acids were found upregulated in RDD, suggesting that the retinal cells may consume alternative energy substrates. The pentose phosphate pathway, besides increasing the production of NADPH, may also have a protective role through the regeneration of reduced glutathione [39]. S-transferase (GSTP1), overexpressed in this study, is an intracellular detoxification enzyme that catalyzes the reduction of electrophiles in retina, iris, and cornea [40,41,42]. Another hypothesis is that increase of carbon metabolism may help to prevent the death of photoreceptors during RRD. The function and survival of photoreceptors and other retinal cells depends on the diffusion of nutrients and oxygen from choroidal circulation [36]. During RRD, this supply is compromised by the physical separation between NSR and RPE, creating an intraretinal environment with starvation of oxygen and glucose. So, the upregulation of enzymes involved in aerobic and anaerobic glycolysis may protect the retinal cells face to hypoglycemia and hypoxia [36,43,44]. Nevertheless, there is some evidence that the glycolytic process is also affected by more severe states of disease, such PVR, where the glycolysis metabolism seems to be significantly reduced [21]. Yu and co-workers found that enolases (ENO2), aldolases (ALDOA, ALDOC), kinases (PGK1, PKM) and other glycolytic proteins (PGAM1, triosephosphate isomerase (TPI1), GPI, LDHB) were significantly down-regulated in moderate PVR [21]. Indeed, some of them disappeared in severe PVR vitreous or were only detected in vitreous from normal human eyes. Other authors found that TPI1 was downregulated in the vitreous from patients with RRDCD [11]. Metabolic analysis of vitreous confirms that the glycolytic profile of vitreous is different between RRD and PVR [45]. Li and colleagues found increased expression levels of d-glyceraldehyde and glycerate in RRD [45], which are metabolites catalyzed by TPI1 and PGK1/PGAM, respectively. These data suggest that glycolysis process is initially upregulated to compensate the metabolic stress of retinal cells after RD, but glycolytic proteins are lost in the progression to more severe conditions. 

The fact that some protective mechanisms are triggered during RRD is also suggested by the upregulation of proteins involved in cellular responses to heat stress and the regulation of apoptotic signaling. Another interesting fact is that many of these proteins are associated with HIF-1 signaling pathway. HIF-1 is a transcriptional regulator that mediates the cellular responses to reduced oxygen levels through changes in gene expression [46,47]. Thus, glycolytic enzymes (ALDOA, ENO2, and PGK1), glucose transporters (SLC2A1), growth factors (TIMP1), and SERPINE1 appear to be upregulated in response to HIF-1. So, HIF-1 may act as a regulator of retinal hypoxia after DDR by controlling cellular anaerobic metabolism, angiogenesis, and cell survival. Proteins such as α-crystallins, β-crystallins, 14-3-3 isoforms and heat shock proteins may also have a protective role in RRD. Despite the role of α-crystallin in retinal and vitreous function has not been fully described [48], most of studies suggest that it has a protective role in degeneration, inflammation, and other retinal stress conditions [49]. Heat shock proteins are a family of proteins that are expressed in response to ocular stress or injury, e.g., ischemia, and participate in folding and repair of damaged proteins [50,51,52]. Particularly, HSP90 seems to have an anti-apoptotic effect mediated by different molecular partners, including the phosphorylated serine/threonine kinase Akt that inhibits the apoptosis though NF-κB (factor nuclear kappa B) [50]. Kayama and colleagues found high levels of HSP70 after RD in mice and rats, which were associated with phosphorylated Akt to avoid its dephosphorylation and further activation of apoptosis [51]. Curiously, other proteins found overexpressed in RRD (PROM1, CHI3L1, YWHAB, CSF1R) were associated with Akt signaling pathways. Specifically, 14-3-3s are small proteins that modulate cell growth and differentiation, regulation of apoptosis and cell cycle. Although the specific role of these proteins in retinal biology is not yet recognized, YWHAQ and YWHAE are the 14-3-3s proteins most highly expressed in the mouse retina and high levels of YWHAE are present in Rod photoreceptors [53]. Curiously, 14-3-3 proteins were found to interact with PDC, after its light-dependent phosphorylation, indicating that these may participate in facilitating the dark adaptation of the photoreceptor [53,54].

Lysosomal enzymes are widely distributed in ocular tissues and their involvement was suggested in the pathogenesis of several diseases, including RD [55]. The RPE is the main responsible for the phagocytosis of photoreceptor outer segments and by its consecutive lysosomal degradation [55,56]. Lysosomal proteins were previously found augmented in the vitreous and subretinal fluid, and their levels were related to RRD duration [57,58]. More recently, higher expression levels of cathepsin D were found in vitreous from patients with PVR [14,21], confirming our results. So, it was suggested that the increase of lysosomal digestion is a later event in RD, contributing to the photoreceptor degeneration and inflammation [57]. Also, considering the role of vitreous liquefaction in RD onset [3,4,7], it has been suggested that lysosomal enzymes, mainly cathepsin D, may be involved in the degradation of glycosaminoglycans, and collagen molecules, of rod outer segments and RHO [55,58,59]. Finally, increased cathepsin A activity in the subretinal fluid was associated with retinal degradation in RD [59]. Another hypothesis is that lysosomal enzymes have a protective effect in the eye, maintaining the health of the neural components of the retina [59].

Phototransduction is a biochemical process essential for vision by which retinal rod outer segment (ROS) capture and convert photons into electrical signals [60,61]. This biochemical cascade is initiated by Rho, a G-protein-coupled receptor found in ROS disks whose structure suffers conformational changes induced by photon absorption [60,62,63]. Transducin is a heterotrimeric G protein that binds the activated Rho, triggering the exchange of GDP by GTP in the α subunit of transducin (GNAT1) and its dissociation from β (GNB1) and γ subunits [60,64]. In its turn, GNAT1 activates phosphodiesterase (PDE6), which is composed of two large catalytic subunits (PDE6A and PDE6B) and two PDE6G subunits, triggering cGMP hydrolysis. Reduction of cGMP levels leads to the closure of the cGMP-gated cation channels in the plasma membrane and to rod cell hyperpolarization [60]. SAG is responsible by regulation of the phototransduction cascade through capture and regeneration of phosphorylated Rho [30]. PDC is a small binding protein found abundantly in photoreceptors that may be responsible for the regulation light sensitivity in the ROS through interaction with the subunits Gβγ of transducin [54]. Considering the specific localization of these proteins in ROS and its relevance to eye function, its accumulation in vitreous implies that the death of photoreceptors occurs after RRD [65,66,67]. The increase of vimentin (VIM) levels found in RRD vitreous also suggest that high levels of retinal stress are induced by the detachment. Vimentin is expressed in retinal astrocytes and Müller cells in the healthy retina but, when RD occurs, the “stress” induces a progressive increase of vimentin in the cell over time, thus becoming the predominant intermediate filament [68,69]. Mandal and co-workers confirmed by 2D-PAGE and immunocytochemistry analysis that the Müller cell hypertrophy is accompanied by an increased expression of intracellular vimentin. This suggests that vimentin and other structural proteins may reinforce the structure of Müller cells in response to RD [63]. Indeed, the lack of vimentin limits the growth of these cells into subretinal space, avoiding the formation of subretinal membrane into this cavity that could jeopardize the photoreceptor regeneration even after successful retinal reattachment surgery. So, this fact can explain the reduced levels of photoreceptor degeneration after RD in in vimentin-deficient mice [70,71]. Another indicator of retinal stress is the highly significant increase (6.5-fold) of the levels of ENO2 in RRD versus MEM, which was confirmed by WB analysis. ENO2 is a cellular damage marker released after retinal neuron injury. High levels of ENO2 were detected in the subretinal fluid, vitreous, and aqueous after RD. In fact, ENO2 appears to be an effective biomarker of retinal damage [72,73]. The maintenance of retinal structure and homeostasis is crucial for healthy vision [74]. Thus, RPE and NSR separation, by reducing the influx of nutrients and oxygen into the retina, induces retinal stress, causing the death of retinal photoreceptors and structural changes in retinal glial cells. 

Although biological events, such as inflammation, immune responses, and coagulation/fibrinolysis have been associated with RD, in this study, only a few proteins related to inflammatory responses were found overexpressed in RRD. Chitinase-3-like protein 1, previously detected in severe PVR vitreous [21], and thrombospondin-1 are positive regulators of inflammatory responses, while SERPINE1 is a negative regulator of fibrinolysis (Supplementary Table S4). Surprisingly, many proteins involved in acute inflammatory response and in complement and coagulation cascades were found underexpressed in RRD. Interestingly, only the components of C1 (C1QC, C1R), the first component of the serum complement system, were found upregulated in RRD. However, previous transcriptomic analysis of human retinal samples reveals that genes related to an inflammatory process are up-regulated in RD, including complement pathway proteins and members of the major histocompatibility complex [5]. So, it is suggested that at the beginning of RRD low levels of plasma proteins are present in vitreous, including inflammatory proteins, complement components, and coagulation factors. This may result from the decrease of the influx of plasma proteins from choroid into vitreous, after the RD, or by the migration of these proteins to the subretinal fluid. It is well known that with the increase of the duration of RRD, the composition of the subretinal fluid becomes more similar to plasma [75]. The blood-retinal barrier (BRB) breakdown, which only occurs later in RD, may also explain the changes in the composition of eye fluids. BRB breakdown and the accentuated levels of inflammatory proteins appear to have a central role in the evolution of RD to more severe pathologies [5,11,22,75]. As a matter of fact, the influx of blood, serum proteins, and vitreal cells through the retinal break is enough to stimulate the PVR development [76]. After the BRB breakdown, the direct influx of cells (RPE cells, fibroblasts, myofibroblasts, among others) into vitreous causes chemotaxis of inflammatory cells [76,77]. For this reason, most of the studies concerning PVR report high levels of plasma protein in vitreous. Albumin, transferrin, apolipoproteins, complement components, members of the serpin family, growth factors and other plasma components were found upregulated in vitreous from patients with PVR [14,21,22,78] and with RRDCD [11]. To fully understanding these findings, it would be extremely relevant to evaluate the variations in the vitreous proteome along the course of the RRD and to compare with other progressive forms of the ocular diseases. In fact, the goal must be to find vitreous biomarkers whose expression levels are correlated with DR severity, i.e., proteins that can act as specific indicators of the disease progression. Although the vitreous levels may better reflect the molecular changes in the eye, the measurement of these biomarkers in body fluids, such as serum or plasma is more accessible for clinical purposes [79]. Therefore, the best strategy is to find the specific disease biomarkers in the vitreous, and, then, try to measure and correlate its levels in serum or plasma. Some authors found a relationship in the levels of kininogen 1 and insulin-like growth factor binding protein 6 between vitreous and plasma in PVR [21,78], but for many other biomarkers, no correlation was found [21,80,81]. In a study of Yu and colleagues, only kininogen 1, among 102 PVR-specific proteins, was specifically detected in both vitreous and serum [21]. Different studies have tried to find a correlation between serum and vitreous in other ocular diseases [82,83,84], including our research group that analyzed the levels of placental growth factor, and vascular endothelial growth factors A (VEGF-A) and B (VEGF-B) [85,86]. In our study, VEGF-A and VEGF-B concentrations were higher in proliferative ocular diseases compared to non-proliferative ocular diseases [86], but no correlation between vitreous vs. serum VEGF-A and VEGF-B was observed. Also, no correlation between vitreous and serum levels of placental growth factor was found in patients with diabetic retinopathy [85]. Comparing to other ocular pathologies, there are few studies in DRR/PVR, which may also explain the lack of biomarkers significantly correlated between serum and vitreous.

## 4. Materials and Methods

### 4.1. Demographics and Clinical Variables

Undiluted vitreous samples were collected via pars plana vitrectomy on the Ophthalmology service of Leiria-Pombal Hospital (Leiria, Portugal), according to the protocol for sample collection approved by the hospital ethics committee (Code: CHL-15481) [87]. An informed consent from all patients was obtained after an explanation of the purpose of this study, which adhered to the tenets of the Declaration of Helsinki. Vitreous samples contaminated with plasma and/or associated with other diseases were excluded, as well as samples from patients subjected to previous intraocular surgeries. The patients had not undergone previous vitrectomies. After exclusion, vitreous collected from 4 patients (1 male, 3 females) diagnosed with RRD were included in the study group, and vitreous collected from 4 patients (2 males, 2 females) diagnosed with MEM were included in the control group. For the validation of iTRAQ results, vitreous from 7 patients were analyzed by WB: 4 patients (2 males, 2 females) diagnosed with RRD and 3 patients (1 male, 2 females) with MEM. Demographic characteristics of patients enrolled in this study and the description of corresponding vitreous samples are summarized in Table 1. Upon collection, vitreous samples were transferred to sterile cryogenic vials and frozen at −80 °C, until further processing.

### 4.2. Vitreous Samples Handling

Vitreous samples were centrifuged at 14,000 rpm for 10 min at 4 °C to separate the soluble proteins from structural components. The protein concentration was determined using a Micro BCA™ Protein Assay Kit (Thermo-Scientific, Porto Salvo, Portugal) and equal volumes of individual vitreous samples were combined according to the study group (RRD vs. MEM), as seen in Table 1. Seppro^®^ IgY14 LC5 and SuperMix LC2 columns (Sigma-Aldrich, St. Louis, MO, USA) were used in tandem for removing abundant plasma proteins from pooled vitreous samples, including albumin and IgG, according to the manufacturer’s instructions. The flow-through fractions were concentrated and desalted using Amicon Ultra-15 3 K Centrifugal Filter Unit (Merck Millipore, Madrid, Spain) and precipitated by chloroform–methanol (4/1, *v/v*). The pellet was resuspended in a buffer with 7 M urea, 2 M thiourea, and 100 mM triethylammonium bicarbonate (TEAB), compatible with iTRAQ labeling. Samples were quantified using RC DC™ Protein Assay (BioRad, Madrid, Spain), according to the manufacturer’s instructions.

### 4.3. In-Solution Digestion and iTRAQ Labeling

After reduction and alkylation, 25 µg of sample was combined with trypsin from porcine pancreas (Sigma-Aldrich) at a final trypsin:protein ratio of 1:10 and digested overnight at 37 °C. Tryptic peptides were dried by vacuum centrifugation, reconstituted in 80 µL labeling buffer (70% ethanol/25 mM TEAB) and labeled with iTRAQ reagents, according to the manufacturer’s protocol (ABSciex, Framingham, MA, USA). Specifically, pooled samples from RRD group (n = 4) and from the control group (n = 4) were incubated with the reagents 116 and 114, respectively, over 2 h at RT. Labeling was confirmed by MS/MS analysis using 4800 MALDI TOF/TOF analyzer (ABSciex, Framingham, MA, USA).

### 4.4. 2D-Nano-LC-ESI-MS/MS Analysis

After labeling, samples were combined and fractionated in an RP column (100 × 2.1 mm, 5 μm particle size, Fortis Technologies, Neston, UK) using a Knauer Smartline HPLC system with UV detection at 214 nm. Peptides fractionation was performed at a flow rate of 150 μL/min with 95% of buffer A (10 mM NH4OH, pH 9.4) for 10 min, followed by a linear increase to 25% buffer B (10 mM NH4OH, 80% of methanol, pH 9.4) for 10 min, to 75% B for 40 min, and, finally, to 100% B. Fractions were collected, pooled into 5 fractions that were dried by vacuum centrifugation and desalted using a SEP-PAK C18 Cartridges (Waters, Milford, MA, USA) [88,89].

Tryptic peptides (5 μL) were desalted onto a trap column C18 PepMap (100 μm × 2 cm, 5 μm, 100 Å, Dionex) using solvent A (0.1% formic acid in water) at 2 μL/min, using an Ultra 2D Plus (Eksigent, Dublin, CA, USA) system coupled to TripleTOF 5600 System via a Nanospray III source (AB Sciex). After desalting, trap column was switched online with an RP nanoACQUITY UPLC analytical column (75 μm × 15 cm, 1.7 μm, Waters). Peptides were eluted at a flow rate of 250 nL/min, using the following conditions: a 110 min linear gradient from 4.8%–30% B (0.1% formic acid in ACN), followed by two linear gradients, 10 min from 30%–40% B and 5 min from 40%–90% B. Two technical replicates were performed for each fraction. TripleTOF 5600 system was operated in positive ion mode with the capillary voltage set at 1500 V, curtain gas of 25 and nebulizer gas of 10. System was operated in an information-dependent acquisition mode with a TOF/MS survey scan (350–1250 *m*/*z*) with an accumulation time of 250 ms. Each MS/MS spectrum was accumulated for 150 ms (100–1800 *m*/*z*) and only the parent ions with a charge state from +2 to +5 were included in the MS/MS fragmentation. Dynamic exclusion allowed that former target ions were excluded for a period of 12 s. The MS/MS spectra were acquired in high sensitivity mode with ‘adjust collision energy when using iTRAQ reagent’ settings.

### 4.5. MS/MS Data Analysis

Raw data files were converted to mgf. files and searched against *Homo sapiens* UniProtKB reviewed database [90] downloaded from Swiss-Prot at 22nd March 2014 and its corresponding reversed database. Database searches were performed using a licensed version of Mascot v.2.2.04 (Matrix science, London, UK). Search parameters were set as follows: enzyme: trypsin allowed missed cleavages: 1; fixed modifications: methythio (C) and iTRAQ4plex; variable modifications: acetyl (Protein N-term), deaminated (NQ) and oxidation (M); peptide mass tolerance: ±25 ppm for precursors and 0.05 Da for fragment masses. 

Relative abundance of the proteins in RRD versus MEM was computed as a weighted average of ratios of the reporter ions (116 vs. 114). Finally, ratios were normalized by dividing each protein ratio by the median value of the tag and the obtained value was log2-transformed. Log2 peptide ratios followed a normal distribution that was fitted using least squares regression. FDR of ≤1% at peptide level was manually assessed using Excel 2010 by applying a target-Decoy approach. Using this strategy, MS/MS data were searched against both the target database and the decoy sequence database, a consciously incorrect database containing reversed shuffled peptide sequences [23]. The peptides identified in this decoy database search result in an incorrect identification, and thus are considered false-positives (FP). Then, FDR is calculated according to the number of FP above a threshold divided by the total number of peptide matches above that threshold. For the selection of differentially expressed proteins, the requirements were (i) identification in both technical replicates, (ii) identification with more than one unique peptide and (iii) the assessed protein ratio was required to be in the 5% most extreme region of a Gaussian distribution fit on all ratios (FDR < 0.05) for both technical replicates. 

### 4.6. Bioinformatic Analysis

Differentially expressed proteins were analyzed according to GO terms for biological process, cellular component and molecular function using STRAP 1.5 (Software Tool for Rapid Annotation of Proteins) at November 2017. To assess functional associations between proteins, differentially expressed in RDD, the online tool STRING 10 was applied with a high confidence (0.70) [91,92]. Protein clusters were defined with MCL clustering using an inflation parameter of 1.3. Pathways enrichment of proteins clusters were performed according to Reactome pathway knowledgebase [93] and KEGG pathway database [94].

### 4.7. Validation by Western Blotting

ENO2, PGAM1, and RHO were randomly chosen from the proteins found differentially expressed in RRD for the validation by WB. Briefly, equal amounts of proteins (15 µg) for each sample were loaded on a 12.5% sodium dodecyl sulfate polyacrylamide gel. Proteins were then transferred from the gel to a PVDF membrane using Trans-Blot Turbo™ Transfer System (Bio-Rad Laboratories, Hercules, CA, USA) for 45 min. After blocking with a solution of 5% of powdered milk in 0.1% Tween-20, the membranes were incubated overnight at 4 °C with monoclonal antibodies prepared in 5% of BSA. The antibodies applied for the validation and respective dilutions are as follows: γ Enolase Antibody (NSE-P1) (sc-21738; Santa Cruz, CA, USA) at 1:500, anti-PGAM1/4 (D-5) (sc-365677; Santa Cruz, CA, USA) at 1:300, and anti-rhodopsin (RET-P1) (sc-57433; Santa Cruz, CA, USA) at 1:300. After the incubation with the primary antibodies, membranes were incubated with an anti-Mouse IgG (Fab specific)–Peroxidase antibody (A3682; Sigma, St. Louis, MO, USA) at 1:10,000. Protein bands were visualized using the Clarity™ Western ECL Substrate (Biorad, Hercules, CA, USA). The detection and relative quantification of the bands was done using Image lab 5.0 software (Biorad, Hercules, CA, USA). Data processing and statistical analyses (Mann-Whitney *U* test, *p* < 0.05) were performed using GraphPad Prism Software (San Diego, CA, USA).

## 5. Conclusions

A total of 1030 proteins were identified using iTRAQ labelling combined with two-dimensional LC-ESI-MS/MS, and 150 proteins were found differentially expressed between RRD and MEM control (96 overexpressed and 54 underexpressed proteins). These proteins were analyzed regarding their molecular function, biological process and KEGG pathways, to better elucidate the molecular mechanism underlying RRD pathogenesis. It is interesting to note that in RRD, there appears to be a balance between death and survival of retinal cells. HIF-1 signaling pathway seems to have a crucial role in the response to retinal stress after RD, promoting the retinal cell survival through the up-regulation of glycolytic enzymes, glucose transporters, and growth factors. The increased levels of molecular chaperones (alpha-crystallins, beta-crystallins) and heat shock proteins (HSP90AA1, HSPA1A, HSPA8) can be related to a protective role in RRD. On the other hand, the accumulation of proteins from photoreceptor cells, NSE, and vimentin in vitreous indicate that the death of photoreceptors occurs in RRD. Lysosomal degradation appears to be up-regulated in RRD, but it is not known whether it has a beneficial or a hazard effect on the survival of retinal cells. Surprisingly, many proteins involved in acute inflammatory response and in complement and coagulation cascades were found underexpressed in RRD. So, processes such as inflammation, coagulation, and fibrosis can be later events in RRD pathogenesis. Vitreous seems to play a key role in the onset of RRD but one must bear in mind that levels of protein in vitreous are an indirect measurement of the events that take place in the retina. Although more studies will be required to fully understand some of these findings, the obtained results provide a basis for new insights in RRD investigation. 

## Figures and Tables

**Table 1:** Demographic and clinical characteristics of patients involved in the study and description of corresponding vitreous samples collected via pars plana vitrectomy.

Demographic and Clinical Characteristics		RRD ^1^ (n = 8)	MEM ^1^ (n = 7)
**Demographic characteristics of patients**	Gender ^2^	M = 3; F = 5	M = 3; F = 4
	Age (MD ± SD)	64 ± 7	76 ± 5
	Age (range)	52–69	69–84
	Eye Submitted to PPV ^3^	LE = 3; RE = 5	LE = 5; RE = 2
**Characterization of retinal detachment**	Macula-off/Macula-in	5/3	
	Extent of retinal detachment (n/n_total_) ^4^		
	1 quadrant	3/8	
	2 quadrants	2/8	
	3 quadrants	2/8	
	4 quadrants	1/8	
	Multiple detachments (n/n_total_) ^4^	4/8	
**Characterization of vitreous samples**	Protein concentration (µg/µL, MD ± SD)	3.12 ± 2.96	2.66 ± 1.63
	iTRAQ label	116 (n = 4)	114 (n = 4)
	Validation by Western blotting	n = 4	n = 3

^1^ RRD: Rhegmatogenous retinal detachment; MEM: Macular epiretinal membranes; ^2^ F: Female; M: Male; ^3^ PPV: Pars plana vitrectomy; RE: right eye; LE: left eye; ^4^ Number of samples/number total of samples.

**Table 2:** List of proteins found differentially expressed in vitreous of patients with rhegmatogenous retinal detachment (RRD) in comparison with macular epiretinal membranes (MEM), with FDR ≈ 0.

Accession	Description	Gene	Score	Number of Peptides (Total/Unique)	Coverage	RRD/MEM Ratio ^1^
P06727	Apolipoprotein A-IV	APOA4	2834	119/3	49.7	0.002 ***
P02753	Retinol-binding protein 4	RBP4	222	10/1	10.4	0.003 ***
O95447	Lebercilin-like protein	LCA5L	25	2/0	2.2	0.003 ***
P50213	Isocitrate dehydrogenase [NAD] subunit alpha, mitochondrial	IDH3A	35	2/0	2.7	0.004 ***
Q96BN8	Ubiquitin thioesterase otulin	FAM105B	28	2/0	2.0	0.005 ***
Q8NBP7	Proprotein convertase subtilisin/kexin type 9	PCSK9	32	2/0	3.2	0.038 ***
P01011	Alpha-1-antichymotrypsin	SERPINA3	1786	63/2	23.4	0.040 ***
P02748	Complement component C9	C9	1126	47/3	13.4	0.058 ***
P02655	Apolipoprotein C-II	APOC2	214	6/2	20.4	0.110 ***
P02656	Apolipoprotein C-III	APOC3	319	10/1	26.3	0.139 ***
Q9HAZ2	PR domain zinc finger protein 16	PRDM16	28	2/0	0.5	0.162 ***
P43652	Afamin	AFM	1336	45/7	13.2	0.162 ***
P02750	Leucine-rich alpha-2-glycoprotein	LRG1	1108	39/5	24.6	0.177 ***
Q6UXB8	Peptidase inhibitor 16	PI16	234	11/3	4.7	0.211 ***
P13646	Keratin, type I cytoskeletal 13	KRT13	440	12/5	16.0	0.230 ***
P35542	Serum amyloid A-4 protein	SAA4	129	4/1	13.9	0.241 ***
Q15166	Serum paraoxonase/lactonase 3	PON3	109	5/0	4.5	0.255 ***
P20941	Phosducin	PDC	47	2/0	5.3	221.22 ***
P14550	Alcohol dehydrogenase [NADP(+)]	AKR1A1	67	2/2	11.1	173.64 ***
P08100	Rhodopsin	RHO	432	15/1	11.1	23.646 ***
P10523	S-arrestin	SAG	1469	56/5	30.5	16.032 ***
P18545	Retinal rod rhodopsin-sensitive cGMP 3,5-cyclic phosphodiesterase subunit gamma	PDE6G	32	2/0	10.3	15.606 ***
P11488	Guanine nucleotide-binding protein G(t) subunit alpha-1	GNAT1	62	2/0	4.3	15.144 ***
Q9UHI8	A disintegrin and metalloproteinase with thrombospondin motifs 1	ADAMTS1	188	4/1	2.2	14.086 ***
O00560	Syntenin-1	SDCBP	496	19/1	20.1	14.012 ***
P11166	Solute carrier family 2, facilitated glucose transporter member 1	SLC2A1	49	3/1	5.1	13.116 ***
Q17R60	Interphotoreceptor matrix proteoglycan 1	IMPG1	1108	35/8	9.5	11.116 ***
O43490	Prominin-1	PROM1	304	11/5	5.4	10.761 ***
P69905	Hemoglobin subunit alpha	HBA1	417	14/3	24.1	10.528 ***
P51674	Neuronal membrane glycoprotein M6-a	GPM6A	133	4/2	7.6	9.061 ***
P62873	Guanine nucleotide-binding protein G(I)/G(S)/G(T) subunit beta-1	GNB1	332	13/6	11.2	8.579 ***
P12277	Creatine kinase B-type	CKB	415	12/5	12.7	8.576 ***
Q9BZV3	Interphotoreceptor matrix proteoglycan 2	IMPG2	302	15/1	4.0	8.451 ***
P16499	Rod cGMP-specific 3′,5′-cyclic phosphodiesterase subunit alpha	PDE6A	243	11/5	2.8	8.274 ***
P43320	Beta-crystallin B2	CRYBB2	775	26/4	37.4	7.078 ***
P62979	Ubiquitin-40S ribosomal protein S27a	RPS27A	247	5/1	10.3	6.897 ***
P68871	Hemoglobin subunit beta	HBB	343	13/1	32.3	6.812 ***
P09104	Gamma-enolase	ENO2	720	24/0	14.4	6.478 ***
P02489	Alpha-crystallin A chain	CRYAA	122	5/1	27.7	6.345 ***
P31025	Lipocalin-1	LCN1	192	7/1	16.1	6.178 ***
P07900	Heat shock protein HSP 90-alpha	HSP90AA1	414	12/3	5.6	6.171 ***
P02511	Alpha-crystallin B chain	CRYAB	108	3/1	22.3	5.648 ***
P02042	Hemoglobin subunit delta	HBD	173	7/2	23.8	5.595 ***
P09467	Fructose-1,6-bisphosphatase 1	FBP1	55	3/1	4.7	5.308 ***
P63104	14-3-3 protein zeta/delta	YWHAZ	455	11/6	10.5	5.127 ***
Q12931	Heat shock protein 75 kDa, mitochondrial	TRAP1	87	3/0	2.0	5.027 ***
P18669	Phosphoglycerate mutase 1	PGAM1	310	12/5	16.6	4.998 ***
P09455	Retinol-binding protein 1	RBP1	50	2/0	8.9	4.680 ***
P36222	Chitinase-3-like protein 1	CHI3L1	1472	55/5	27.1	4.635 ***
Q06830	Peroxiredoxin-1	PRDX1	234	9/3	8.8	4.531 ***
P37837	Transaldolase	TALDO1	116	5/3	10.4	4.388 ***
P09972	Fructose-bisphosphate aldolase C	ALDOC	818	28/6	24.3	4.286 ***
P31949	Protein S100-A11	S100A11	38	2/0	8.6	4.258 ***

^1^ Fold changes >1 are considered for overexpressed proteins and <1 for underexpressed proteins with significant differences (*** *p*-value < 0.0001) between RRD and MEM.

**Table 3:** Comparison of proteins identified in vitreous using different experimental set-ups.

Experimental Set-up	Number of Identified Proteins ^1^	Number of Proteins Exclusively Identified	Reference
HAPs depletion 2D-LC-MS/MS (TripleTOF 5600)	1030	222	Present study
HAPs depletion IEX, SDS-PAGE, MALDI-TOF/TOF	127	63	[19]
CE-MS (micro-TOF MS)	101	-	[35]
CE-MS (micro-TOF MS)	94	-	[33]
2D-LC-MS/MS (LTQ Velos)	1575 ^2^	653 ^2^	[34]
RP-LC-ESI-MS/MS (Orbitrap Elite hybrid MS)	2482	1696	[32]
CE-MS (micro-TOF MS)	96	-	[31]
HAPs depletion SCX, SDS-PAGE, and OFFGEL, RP-LC-MS/MS (LTQ-OrbitrapVelos)	1201	324	[30]
SDS-PAGE and IEF, RP-LC-MS/MS (LTQ-Orbitrap XL MS)	1110	302	[29]
SDS-PAGE, LC-MS/MS (LTQ)	249	13	[28]
HAPs depletion 2DE, MALDI-TOF SDS-PAGE, LC-MALDI-TOF/TOF, and LC-MS/MS	455	54	[27]
2DE, LC-Q-TOF/TOF (QTOF2)	13	-	[26]
SDS-PAGE, MALDI-TOF	12	-	[25]
2DE, LC-Q-TOF/TOF, and MALDI-TOF	18	-	[24]
2DE, MALDI-TOF, and LC-MS/MS (LCQ DECA) IEX, LC-MS/MS (LCQ DECA)	54	19	[23]

^1^ In all these studies, protein isoforms were referred as a single protein; ^2^ Only non-redundant proteins were considered.

## Supplementary Materials

Four supplementary tables are provided as supplementary materials at http://www.mdpi.com/1422-0067/19/4/1157/s1. Table S1.1—Protein list corresponding to proteins only identified in technical replicate 1 and 2 with an FDR of 1% at the peptide level. FDR and p-value displayed in the table are related to quantification analysis using iTRAQ labeling and not to protein identification. Proteins highlighted in gray, red and green correspond, respectively to the non-differentially expressed, underexpressed and overexpressed in RRD (116) when compared with MEM control samples (114). Table S1.2—Peptide list corresponding to proteins identified in technical replicate 1 and 2 with an FDR of 1% at the peptide level. Cells highlighted in light pink and blue correspond to peptides identified in replicates 1 and 2, respectively. Unique peptides are highlighted at green. Table S2.1—Comparison of published proteins found in vitreous humor proteome in previous reports with the list of proteins identified in the current study. Table S2.2—List of proteins of vitreous humor proteome newly identified in the current study. Table S3.1—Proteins found differentially expressed, which were identified in technical replicate 1 and 2 with a FDR of 1% at the peptide level. FDR and p-value displayed in the table are related to quantification analysis using iTRAQ labeling and not to protein identification. Proteins highlighted in gray, red and green correspond, respectively to non-differentially expressed, underexpressed and overexpressed proteins in RRD (116) when compared with MEM control samples (114). Table S3.2—Protein list corresponding to the proteins found differentially expressed, which were identified in technical replicate 1 and 2 with a FDR of 1% at the peptide level. FDR and p-value displayed in table are related to quantification analysis using iTRAQ labeling and not to protein identification. Proteins highlighted in gray, red and green correspond, respectively to non-differentially expressed, underexpressed and overexpressed proteins in RRD (116) when compared with MEM control samples (114). Table S4.1—Results of STRAP bioinformatics analysis of differentially expressed proteins found in vitreous humor proteome of RRD patients compared with MEM control samples. Proteins are classified according their function, catalytic activity and gene Ontology (Go terms for biological process, cellular component and molecular function. Table S4.2—Classification of differentially expressed proteins found in vitreous humor proteome of RRD vs MEM according to biological processes using STRAP. Table S4.3—Classification of differentially expressed proteins found in vitreous humor proteome of RRD vs MEM according to molecular function using STRAP. Table S4.4—Clusters in the protein-protein interaction network of the proteins found differentially expressed in RRD. The clusters were predicted using the ECM clustering option provided by STRING. Table S4.5—Pathways enrichment of proteins clusters according to KEGG pathway database. Table S4.6—Pathways enrichment of proteins clusters according to Reactome pathway knowledgebase.

## Abbreviations

2D-LC-MS/MS	Two-dimensional liquid chromatography-electrospray tandem mass spectrometry
AKR1A1	Alcohol dehydrogenase [NADP(+)]
ALDOA	Fructose-bisphosphate aldolase A
ALDOC	Fructose-bisphosphate aldolase C
APOA4	Apolipoprotein A-IV
APOC2	Apolipoprotein C-II
APOC3	Apolipoprotein C-III
BRB	Blood-retinal barrier
C1R	Complement component 1
C8A	Complement component 8
C8B	Complement component 8
C9	Complement component 9
ENO2	Enolase 2
F12	Coagulation factor XII
F9	Coagulation factor IX
FBP1	Fructose-1,6-bisphosphatase 1
GO	Gene ontology
GPI	Glucose-6-phosphate isomerase
GSTP1	Gstp1
HIF-1	Transcriptional regulator hypoxia-inducible factor-1
iTRAQ	Isobaric tags for relative and absolute quantitation
KEGG	Kyoto encyclopedia of genes and genomes
LC-ESI-MS/MS	Liquid chromatography-electrospray ion trap-mass spectrometry-mass spectrometry
LDHB	L-lactate dehydrogenase
MALDI-TOF/TOF	Matrix-assisted laser desorption/ionization-tandem time-of-flight mass spectrometry
MEM	Macular epiretinal membranes
NSR	Neurosensory retina
PDC	Phosducin
PDE6G	Retinal rod rhodopsin-sensitive cgmp 3~,5~-cyclic phosphodiesterase
PGAM1	Phosphoglycerate mutase 1
PGK1	Phosphoglycerate kinase 1
PKM	Pyruvate kinase
PVR	Proliferative vitreoretinopathy
RBP1	Retinol-binding protein 1
RBP3	Retinol-binding protein 3
RBP4	Retinol-binding protein 4
RD	Retinal detachment
RD	Retinal detachment
RHO	Rhodopsin
RLBP1	Retinaldehyde-binding protein 1
ROS	Retinal rod outer segment
RPE	Retinal pigment epithelium
RRD	Rhegmatogenous retinal detachment
RRDCD	Rhegmatogenous retinal detachment associated with choroidal detachment
SAG	S-arrestin
SLC2A1	solute carrier family 2, facilitated glucose transporter member 1
STRAP	Software Tool for Rapid Annotation of Proteins
STRING	Search Tool for the Retrieval of Interacting Genes/Proteins
TALDO1	Transaldolase
TPI1	Triosephosphate isomerase
VEGF-A	Vascular endothelial growth factor A
VEGF-B	Vascular endothelial growth factor B
WB	Western blotting
